# Foreign Bodies in the Eye

**Published:** 1847-03

**Authors:** 


### SURGICAL SOCIETY OF IRELAND.

*Foreign Bodies in the Eye.* By Dr. Jacob.—Professor Jacob begged to bring under the notice of the society a small matter of which he had no written statement, but which he considered not destitute of pathological interest. The specimen he had to show was one of a foreign body projected into the eye, which he remarked was not unique, for some cases of the kind were on record. In breaking or dressing stones, it frequently happens to stone-cutters and others that a particle of the stone is driven with considerable force into the eye. This it was that happened in the case to which he was about to direct the attention of the society. A particle of stone had been so projected, and lay in the anterior chamber between the cornea and iris, but the interesting fact connected with this was that it should have remained in that situation for four years without having effected the destruction of the organ. He had extracted it the other day, and had every hope that he would ultimately be able to save the eye. The lens is opaque, and the pupil eccentric, and it will probably be yet necessary to break up the lens more effectually than could have been done in the course of the operation of removing the foreign body. In cases of this kind, those men most often suffer who are employed in dressing mill-stones, cutting or breaking siliceous rocks; such accidents seldom occurring from cutting granite or lime-stone. The fragment in the present case was at least a fourth of an inch long and a sixth in diameter, and very sharp. Cases in which foreign bodies of this description had passed into the eye without destroying it, have (Professor Jacob observed) been recorded by Mackenzie, Lawrence, Wardrop, and others, so that, as he had before remarked, there was nothing very new in the case now before the society, but its pathological interest he considered of the first importance, showing as it does that when a foreign body of such a description finds its way into, and remains for such a lengthened period, in an organ of all others in the body so profusely supplied with nerves and vessels without causing its destruction, it need not to be considered necessary to search with such anxiety after foreign substances that find their way into other and so much less important parts of the body, under the apprehension that they will make their way eventually to the surface, instead of which they often remain at rest after a little time, if the part be kept quiet.

Another case had come under his care some years ago: it was that of a little boy into whose eye a portion of a copper gun-cap had passed through the pupil, and lodged in the crystalline lens itself, where it lay without producing any distress or mischief for two or three years. But the very curious fact connected with it was, that the copper never lost any of its metallic brilliancy, and never became even in the slightest degree corroded or oxidated. This case he temporized with, and the sequel proved very instructive; the lens became absorbed, and the bit of copper got entangled in the opaque capsule, and believing that it might not be possible to extract it, the patient being young and unmanageable, he still continued to temporize with the case, and lost sight of the boy for some time.

In about a year after, however, he again came under his notice, and now the copper cap had disappeared, and the anterior and posterior chambers were filled with blood, as if from some recent injury. The pupil was dilated, but the eye was spoiled. The cap being nowhere visible, it was probable that it had fallen to the bottom of the eye, so Professor Jacob considered it better to leave the eye alone, and the case was lost sight of. It is not alone in the anterior chamber, or in contact with the iris or crystalline lens, that bodies of this kind stick, but sometimes under the conjunctiva itself, though (Professor Jacob observed) from the toughness of the parts, it did not often happen; this, however, occurred in the case of a young lady, whose younger brother, in playing with a toy-gun, drove a portion of the cap into her eye, where it lay under the conjunctiva, its situation being indicated by a small blackish tumour underneath that membrane. Having felt the little body with the point of a needle, he was enabled to remove it with the scissors without difficulty. In this case the foreign body had lain for nine months without producing any material mischief. Many instances are recorded in which bits of straw, pieces of rush, twigs, and such matters, become impacted, and where (Professor Jacob observed) one would never suppose they could lie quietly for any time, and that not alone beneath the conjunctiva, but in the fold of reflection of this membrane to the upper lid. Here they sometimes lie without attracting any attention until the production of a fungous tumour over the foreign body brings the case under the notice of the practitioner. A gentleman presented himself under circumstances of this kind to him, and on attempting to remove the tumour with a fine pair of scissors, he found that he could not do so in consequence of some hard unyielding material, which he extracted after snipping off the tip, and which proved to be a portion of the flowering part of a rush half an inch long, which had probably been driven into the part some months before ; the patient having had a fall from his horse in the field about that time.

Now, (Professor Jacob observed) the question suggests itself as to how cases of this kind are to be dealt with ; should they be temporized with ? This he considers we are perfectly justified in doing if the body has lain quietly for a considerable time, and more especially, if it be exceedingly small ; but if the foreign body is the source of a certain amount of irritation, the distress experienced will of course compel the surgeon at once to remove it. In the case first alluded to, and a specimen of which had been sent round, there had been, as he had stated, a great deal of inflammation, followed by an eccentric pupil and opaque lens, yet no shrinking of the eyeball or other indication of destructive inflammation. With respect to the mode of procedure for the removal of foreign bodies in the interior of the eye, Professor Jacob would recommend the method pursued in the case of the fragment in the anterior chamber, so little effusion or distress had followed upon it. In such a case it is supposed that it is only necessary to make an incision in the cornea, and let the foreign body pass out as the lens would in extraction ; the operation is, however, attended with greater difficulty than might at first sight be expected.

In the first place, if recently lodged in the eye, a good deal of difficulty attends its extraction. It is stated by Lawrence, Tyrrel, and others—though he (Professor Jacob) was not satisfied of the fact—that these bodies become adherent, that they become imbedded or enveloped in lymph. This, to be sure, one might expect, arguing from what occurs in other parts of the body under similar circumstances, but such was not the result of his experience ; for instance, in the case under consideration, the particle of stone was perfectly clean and as distinctly visible in the anterior chamber, as if placed for examination in a drop of water. There was positively no lymphy coating either on the stone or in the case of the copper-cap ; indeed, he could not clearly see where the lymph was to come from ; if lymph were shed, it would of course be the result of inflammatory action, and the foreign body would become adherent in consequence.

He would not, as he had said, deny that such might have occurred in other hands, but it was not so with him, and perhaps the statement was made by others under the conviction that a foreign body could scarcely remain in such a situation without becoming encysted. At the same time in the operation of extracting such substances it is well to be aware that they may be and probably often are adherent. In proceeding to extract them, as a general rule he would say, that the incision should be as large as possible, for the difference of mischief to the eye, between a small and large incision, is as nothing compared with the difficulty of dragging the foreign body through a small orifice. This will more particularly be necessary if the foreign body be a stone, as happened in the case now described, in which it was only after a second or third trial that Professor Jacob was enabled to extract it. Having waited between each attempt at extraction for the patient to become composed, and the spasmodic action of the muscles to subside, he was at length enabled to remove the fragment with the aid of the curette, and not without a good deal of force employed in disentangling it, for the little body was no doubt adherent, though not at all imbedded or encysted. Notwithstanding a good deal of violence having necessarily been employed, the case is going on well, there being no inflammation, such as would lead to the destruction of the organ. The man has already so much vision as to satisfy Professor Jacob that ultimately the eye will enjoy a useful amount of it. At the stage of his remarks, Professor Jacob said, it became a question for consideration whether in cases of this kind seen immediately after the accident, the practitioner would proceed at once to the removal of the foreign body. By all means, he would say, if he sees the case in the course of the day upon which the mischief has been done, it ought, if possible, to be removed at once, and this may sometimes be effected with the greatest facility, while in other cases the greatest difficulty is encountered. Thus it will happen that to remove a body lying in the anterior chamber there is nothing to be done but to pass the knife, cut through the edge of the cornea, and the foreign body immediately drops out; but in other instances, in spite of the utmost caution, it falls backwards into a fold of the iris, and entirely disappears from view; following it, however, with the curette, the extraction may be effected, notwithstanding that the lids are squeezed up, and every effort made by the patient to turn the eye away from the operator. In this college, many years ago, (Professor Jacob said) a case of this kind occurred in a pupil of the institution, who, while at work in the laboratory, had a splinter of glass driven into his eye from the bursting of a glass vessel. The particle passed through the cornea, and on seeing him immediately after, Professor Jacob observed the bit of glass lying in the anterior chamber, with one point resting against the cornea, and the other against the iris. Having made a considerable opening with the extracting knife, the foreign body almost disappeared at first, having fallen into the fold of the iris, but with the curette he was fortunately enabled to lift it up, and the extraction was made without any subsequent injury to the organ. This case occurred in a gentleman who is at present a fellow of the college.

Professor Jacob next alluded to the minute foreign bodies which so frequently become fixed in the cornea, and which cause so much trouble to the practitioner in this country. where stone-cutting, quarrying, and stone-breaking is so common ; and when, as elsewhere, so many working at the anvil, bench, and lathe, are liable to such accidents.

The foreign bodies in these cases (Professor Jacob observed) are either particles of the steel of the instruments used by the workmen, or portions of the stone itself, but in nine cases out of ten they are particles of the steel, and it becomes of importance to ascertain in what state the steel is at the time. As regards the difficulty of removal, a great deal depends on the size of the particle, which is sometimes wonderfully minute, so much so as to become scarcely visible after a great deal of trouble and minute examination, and occasionally only with the assistance of a lens of two and a half inch focus, after twisting and turning the eye up and down, in and out, in all directions. Yet so small a particle will be productive of the greatest mischief. In general those particles may be removed with great ease, requiring only to be touched with the point of a blunt instrument very cautiously, such as the handle of a camel-hair pencil, pared down very fine, or the convex part of his own cataract needle. If only adherent to the conjunctival layer, the particle slips off by the gentlest means, and if that does not succeed, or if the foreign body is more deeply imbedded, if it has got into the structure of the cornea, its removal is more difficult. If the foreign body has been projected with violence, and has become imbedded in the cornea itself, it must be lifted out of it. The point of the needle should be held within a very short distance of the foreign body before it touches the cornea, waiting quietly until the eye becomes steady; it should then be struck in beneath it, and the particle dug up, if not detached, by gentler means. The operator should never give up until he has fairly lifted it from its situation. Many surgeons at the other side of the water—among whom is so high an authority as Mr. Lawrence—recommend such cases to be left to nature, saying that a little spot of ulceration is formed round the foreign body which thus becomes washed away by the secretion of the tears ; but this ulcer will ultimately leave behind it an opacity. As regards those cases in which small particles of steel have been projected into the cornea, and the particles so dissolved away that only a small portion of the oxide adheres to a speck of ulceration, this is to be removed with a few touches of the point of the needle, so as to prevent the occurrence of any permanent opacity. But of all circumstances connected with this subject (Professor Jacob observed) that which is of paramount importance to the practitioner refers to the condition of his patient's general health at the time of the accident. In this country these accidents to the eye are in themselves generally trivial, but the worst results sometimes occur to the subjects of them from an unhealthy state of the constitution. He had known total blindness to occur in stone-breakers from this cause much oftener than would be believed. Even when the foreign body is got rid of a destructive inflammatory process is set up under these circumstances which ends in the loss of the eye. A whitish sloughy ulceration takes place in the injured part, purulent matter is deposited in the anterior chamber, and at last the sloughy ulceration extends through the cornea.

To prevent or remedy this, attention must be paid to the state of the digestive organs and health in general. A yellow coated tongue is a sure indication of that state of constitution in general, and gastric organs in particular, which leads almost with certainty to this state of things, and this must be remedied by the usual medicinal and dietetic remedies, recollecting that such cases seldom bear or require depletion.

The learned professor concluded by apologizing for the length of time that he had occupied the attention of the society. He would, he said, not have gone so much in detail upon the subject, but that there are so many practical points connected with it, not always to be found in books, and upon which he had hopes of eliciting observations from the gentlemen present, which would throw additional light on the subject.

Mr. M'Coy mentioned the case of a young female who had come under his care for a painful abscess of the lower jaw. While examining her face he observed a bright silver-like looking body adhering to the iris. Her mother stated her age to be eighteen, and that the speck alluded to had always been in the eye, at least since she was a child; on further inquiry, however, it appeared that many years ago, while playing tricks on Hallow Eve, a particle of melted lead had flown into the eye, which latter became closed for two or three days, but no further inconvenience resulted from the accident. There remained no trace of wound or injury of the cornea, nothing but the brilliant little body just described, whose measurement was about a line in one way and half a line in the other. The circumstance (Mr. McCoy observed) appeared remarkable in two points of view—first, that the substance at the moment of its penetrating the cornea must have been very hot, yet upon examination there was not the slightest trace of lymphy exudation into the corneal structure; secondly, the body having for such a length of time retained its brilliancy.

Professor Jacob.—What Mr. M'Coy has just stated, one would at first sight say was a very improbable occurrence—namely, the projection of a particle of lead into the eye without producing great destruction. Again, the great improbability of a particle of such a body as lead being projected with such a degree of violence as to penetrate the cornea, and be deposited in the anterior chamber. Yet he was inclined entirely to agree with Mr. M'Coy as to the facts of the case. Particles of melted lead thrown upon water are sometimes cast up with great force, and he had known instances in which children have had particles of melted lead thrown into the eye and retained beneath the lid, while persons were unaware of their presence there. In one such case, were a good deal of distress and mischief had been produced, he had removed after some weeks a portion of lead which had evidently found its way under the lid in a melted condition.

Dr. Hargrave wished, while engaged on this subject, to ask Professor Jacob what means he was in the habit of using for examining the fold of conjunctiva lying between the globe of the eye and upper lid. He recollected the case of a young man in respectable life who had been treated very actively for acute inflammation of the eye, and who maintained himself that a particle of the bearded portion of barley had got into his eye, but on the most careful examination nothing of the kind could be detected. On seeing him some time after, there was still a good deal of distress, accompanied by symptoms resembling chronic inflammation, and the patient still insisted on the presence of the foreign body. Dr. Hargrave could scarcely credit this statement, knowing how accurately he had searched for it before, yet upon again examining closely, a bit of the bearded barley, half an inch in length, was found completely impacted in the fold of conjunctiva just alluded to. The great difficulty of examination in these cases arises from the spasmodic action of the orbicularis muscle, which was found to be equally uncontrollable either in the very young, the middle aged, or the old. He would beg to express his admiration of the truly practical and valuable remarks of Professor Jacob on the subject under discussion.

Professor Jacob said the suggestion made by Dr. Hargrave respecting the impaction of foreign bodies and the difficulty of their removal from the situation indicated, was a very important one. Examples of the kind are recorded by Scarpa, Monteith, &c., and a case is mentioned by Mackenzie in which a portion of straw had become impacted in the sulcus alluded to. By the same author the case of a child is mentioned in whose eye a common fly had got entangled, followed by a great deal of mischief. He would say that the most feasible way to remove such would be to carry the curette slowly round between the eyeball and orbit. In such a case he thought it would be better not to evert the lid. By a little nice and dexterous management on the part of the surgeon, he was of opinion that even the smallest specks could be detected and removed.

Dr. Bigger suggested a mode of removing foreign bodies (not impacted) in this situation. The method is one proposed by an authority who has written on this subject, and by whom it had been successfully applied on many occasions. It is simply this: on raising the upper lid which conceals the foreign body, the struggles of the patient cause the lashes of the lower lid to approximate the globe, and the foreign body falling down becomes entangled in them, and thus carried out of the eye. Dr. Bigger had frequently tried this method with success in children, but of course if the body be impacted, additional means of extraction will be found necessary.

Professor Apjohn observed in reference to one particular point alluded to in the discussion of this very practical subject—viz., the preservation of their natural brilliancy by the little metallic bodies projected into the eye in those cases, that the explanation appeared to him to be as follows. The liquid in which the foreign body is immersed is totally devoid of free oxygen—it is, in fact, alkaline. Thus the body lying in a fluid which, unlike common water, contains neither air nor oxygen in a gaseous condition, maintains its characteristic lustre. If a piece of metal were placed in a vessel hermetically sealed, so as entirely to expel the air, its brilliancy might be preserved for any length of time, and the bit of metal in the cases alluded to might be looked on as placed very much in that predicament.

The Vice President expressed his admiration of the very valuable communication made by Professor Jacob; the observations which fell from that gentleman were, he said, ten times the more valuable because they were of such a nature as cannot be conveyed in books. He (Mr. Cusack) felt desirous to know Professor Jacob's opinion upon the difference in the state of oxydation of these metallic particles as seated in the interior of the eye or only under the conjunctiva; for it appeared to him that in the latter case the thinness of the mucous membrane rendered it perhaps not altogether impermeable to atmospheric air. For instance, many years ago it had happened to him in the case of a person admitted to the hospital for chronic inflammation of the eye, when turning up the lid for the purpose of examination, to observe something black in the corner of the eye. Examination with a probe proved it to be a metallic substance, and it appeared to Mr. Cusack, when pressed on, to be lodged in the substance of the eye itself. The history was as follows:—The man had some time before had, in a scuffle, a fork thrown at him, which consisted of but one prong. This it appears having passed through the eye, was broken off, leaving the small portion, which was now visible, projecting at the inner canthus and edge of the upper lid. It was difficult (Mr. Cusack remarked) to conceive how the fork should have taken such a direction, unless we suppose the person hit to have been placed in a position above that of the aggressor, and it is with this supposition only that we can account for the orbital plate of the frontal bone having escaped penetration under the circumstances. Another circumstance in Professor Jacob's remarks must (Mr. Cusack observed) have struck the students amongst the audience as a very peculiar feature in connection with this subject—namely, the intense sensibility of the conjunctival membrane and the acute attacks of inflammation it undergoes from comparatively trivial causes, while the eye itself can be subjected to so much violence. As an example of this intense degree of sensibility on the part of the conjunctiva, Mr. Cusack mentioned the case of a gentleman of rank into whose eye a fly had passed, and got impacted under the conjunctiva; this was a source of suffering for a length of time until discovered by a practitioner of acute observation.—*Dublin Medical Press.*

